# HiCognition: a visual exploration and hypothesis testing tool for 3D genomics

**DOI:** 10.1186/s13059-023-02996-9

**Published:** 2023-07-05

**Authors:** Christoph C. H. Langer, Michael Mitter, Roman R. Stocsits, Daniel W. Gerlich

**Affiliations:** 1 Institute of Molecular Biotechnology of the Austrian Academy of Sciences, Vienna BioCenter, Vienna, Austria; 2 Research Institute of Molecular Pathology, Vienna BioCenter, Vienna, Austria

## Abstract

Genome browsers facilitate integrated analysis of multiple genomics datasets yet visualize only a few regions at a time and lack statistical functions for extracting meaningful information. We present HiCognition, a visual exploration and machine-learning tool based on a new genomic region set concept, enabling detection of patterns and associations between 3D chromosome conformation and collections of 1D genomics profiles of any type. By revealing how transcription and cohesion subunit isoforms contribute to chromosome conformation, we showcase how the flexible user interface and machine learning tools of HiCognition help to understand the relationship between the structure and function of the genome.

**Supplementary Information:**

The online version contains supplementary material available at 10.1186/s13059-023-02996-9.

## Background

Regulated expression, maintenance, and propagation of the genetic information depends not only on the DNA sequence but also on the thousands of different proteins and posttranslational modifications that enrich at specific sites of the genome. The regulation and function of genomes further depends on an intricate organization of DNA in 3D space [1, 2], established by DNA looping [3], chromatin phase separation [4–6], and potentially other processes. How 3D genome organization relates to local variation in chromatin composition, DNA sequence, and physiological functions are key questions that will be important to answer for understanding the function of complex genomes.

The advent of techniques mapping function, composition, and 3D organization genome-wide provides rich sources of complex data to address this challenge. Curated public repositories of various functional and 3D genomics data, e.g., Encyclopedia of DNA Elements (ENCODE) [7, 8] and 4Dnucleome [9], provide opportunities for experimentalists to assess their data in the context of multi-dimensional epigenetic and spatial signatures. However, the challenge of extracting meaningful information from large sets of complex data has hampered progress.

A common approach towards identification of biologically relevant patterns is by studying relationships between multiple independent experiments, representing different assays, molecular components, cell states, or treatments. For example, the observation that the protein complex cohesin enriches at insulation sites of transcriptional regulation [10] and at the boundaries of topologically associated domains (TADs) [11] has inspired models for how the genome is organized by cohesin-mediated loop extrusion [12–14], with broad implications for various processes [3]. Detecting associations between multiple genomics datasets is facilitated by genome browsers [15–18], which provide side-by-side views of functional genomics data and support user interaction by panning and zooming. However, currently available genome browsers visualize only a small number of regions at a time, which restricts the assessment of large genomes and highly heterogeneous signals in genomic profiles. To facilitate visualization and grouping of small multiples of genomic regions, a set of tools has been recently developed to leverage the concept of visual piling [19, 20]. While these tools allow detection of patterns in single genomic tracks, they do not support integration of different data sources and have performance limitations with large sets of genomic views.

Systematic analysis of correlations in multiple independent genomics datasets often starts by defining a specific type of genomic region based on a common function (e.g., genes) or experimental observation (e.g., ChIP-seq peaks). Owing to the necessity to interface different data types and to combine algorithms from different sources, the analysis of genomic region sets is typically performed by script-based approaches [21–23]. While script-based analysis provides flexible access to powerful statistics and machine learning tools [24–26], it often takes a lot of time and requires advanced programming expertise to adapt workflows for investigation of new biological questions. Many wet-lab biologists have limited expertise in scripting or programming and therefore delegate advanced data analysis tasks to dedicated computer scientists, which represents a severe bottleneck in testing and developing new hypotheses.

Here, we present HiCognition, a tool for interactive visualization and statistical analysis of 3D genomics data and other (epi)genetic profiles based on a region set concept. HiCognition combines a visual exploration interface with high-performance data processing and statistical and machine learning tools. Thereby, HiCognition allows biologists without programming skills to systematically explore their large multi-dimensional genomics data, providing unprecedented opportunities for discovering fundamental mechanisms underlying the organization and function of the genome.

## Results

### Exploring genomic region sets in multi-dimensional feature space

In contrast to conventional 3D genome browsers like JuiceBox [17] or HiGlass [16], which visualize a specific subregion of the genome that can be panned or zoomed, HiCognition has been designed for interactive analysis of large sets of genomic regions that are pre-defined by the user before data exploration. The *genomic region set* approach of HiCognition allows users to address biological questions about how a specific type of region is composed, regulated, and organized in 3D space. The genomic region set can be freely defined by the user, for example, based on a common function (e.g., genes, enhancers, or origins of replication), based on molecular composition (e.g., regions with specific histone modifications or enrichment sites of proteins), or based on 3D organization (e.g., loops or topologically associated domains). The region set can be directly imported into HiCognition from the public repositories ENCODE [7, 8] and 4D nucleome [9] or provided as a file containing genome coordinates. HiCognition then allows the user to explore associations between the genomic region set and large collections of genomics features, which can also be directly imported from public repositories or as files from lab-internal experiments.

In HiCognition, *genomic features* can contain any type of numerical data associated with genomic coordinates [27–29], including two-dimensional data like chromosome conformation contact maps (e.g., from Hi-C [30] or SPRITE [31, 32]), or one-dimensional data such as protein binding profiles (e.g., ChIP-seq [33] or Cut&Run [34] read densities), chromatin accessibility measurements (e.g., ATAC-seq [35] or MNase-seq [36]), transcriptional activity (e.g., GRO-seq [37]), or replication timing measurements (e.g., Repli-seq [38]). Moreover, genomic features can contain data from unperturbed conditions as well as data obtained after genetic or chemical treatments, or data from different cell states (e.g., cell cycle stage or differentiation state), thereby enabling queries of how specific types of regions respond to perturbations or state transitions. HiCognition combines an intuitive and configurable graphical user interface with statistics and machine learning methods to enable interactive exploration of multi-dimensional genomics data within versatile workflows.

**Fig. 1:**
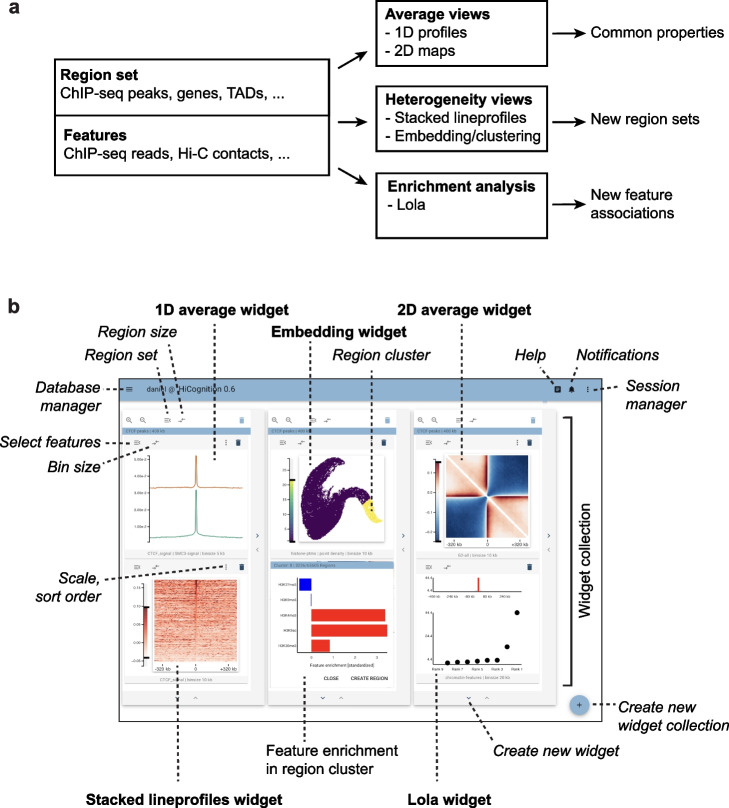
HiCognition concept and graphical user interface. **a** Analysis workflows based on genomic region sets and collections of 3D genomics/epigenetic profiles. **b** Graphical user interface with freely configurable widget layout. Widgets are labeled in bold and user interface elements are marked in italic. Widget collections in this figure represent visualizations of different properties of the same region set (genome-wide set of CTCF ChIP-seq peaks) with various ChIP-seq or Hi-C datasets; for explanation of individual widgets and data, please see Figs. 2 and 3 and main text

HiCognition supports data analysis by three basic approaches (Fig. 1a):

1. *Exploring average distributions:* HiCognition visualizes average magnitudes of genomic signals within the region window, whereby the features can be interactively selected by the user.
2. *Exploring region heterogeneity:* HiCognition visualizes genomic signals of individual regions to visually explore heterogeneity in the region set. Moreover, multi-dimensional cluster analysis and visualization of region distributions in embedding plots allows identification of region sub-sets with common properties.
3. *Enrichment analysis:* HiCognition automatically detects features that are enriched or depleted in the specific region set under investigation relative to the genome-wide average. It further shows where, within the genomic region window, individual features are particularly enriched or depleted. This enables the discovery of regulatory, functional, or spatial patterns characteristic for the region set under investigation.

The user interface of HiCognition is based on a widget architecture that allows easy configuration of views. These widgets represent genomic features and are arranged within widget collections that are associated with a specific genomic region set (Fig. 1b). This arrangement maps the abstract region set concept to a specific user interface component, allowing users to construct views that integrate different genomic features to understand the properties of a genomic region set. Specifically, following import and pre-processing of region and feature datasets, HiCognition widgets generate average feature signal plots of all regions, as well as stacked representations of individual regions, whereby the graphical user interface allows interactive adjustment of region size, resolution, look-up table, contrast, etc. For automatic detection of genomic features enriched in the region set, HiCognition provides a widget for locus overlap analysis (LOLA [39]), which is displayed as a ranked feature plot. For the analysis of heterogeneity within the region set, a clustering and embedding widget automatically groups regions based on similarity in multi-dimensional feature space and represents their distribution in embedding plots. The embedding plots are interactive and display feature patterns for individual region clusters to allow fast, interactive exploration of heterogeneity within the region set. Overall, this widget architecture with interactive visualization integrates improved versions of domain-specific tools [39] and creatively applies state-of-the-art machine learning for embeddings [40] and clustering.

HiCognition is implemented as a web-based tool that allows performant analysis of large datasets and interactive exploration of aggregation results. The software is open source and fully containerized, such that it can run on centralized servers or locally. An integrated database for region sets and features makes HiCognition a hub for various data types from public or private sources, whereby a session concept allows sharing of insights as fully customizable views and analysis workflows with others. A public server instance of HiCognition along with example data for hands on experience can be freely accessed at https://app.hicognition.com/.

### Revealing common patterns in region sets

**Fig. 2:**
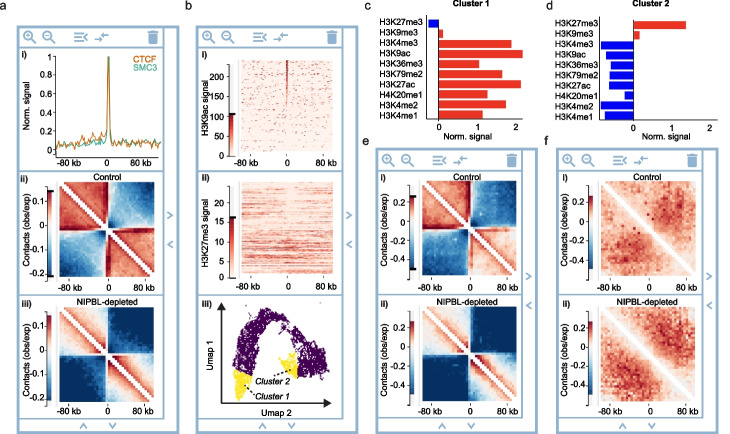
Exploring average profiles and heterogeneity in region sets. A genome-wide region set of protein-coding genes was analyzed based on published ChIP-seq and Hi-C data, centered on the TSS. Data show averages of genes localized on the forward strand, with the gene body facing towards the right from the TSS (center coordinate). **a** Average ChIP-seq reads of CTCF and SMC3 (i), average Hi-C contact maps of unperturbed wildtype cells (ii), and average Hi-C contact maps of NIPBL-depleted cells (iii). **b** Heterogeneity of histone posttranslational modifications within the protein-coding gene region set, visualized by stacked line profiles sorted by the read density of H3Kac (i) and displayed for H3K27me3 ChIP-seq read density (ii), with the sorting order coupled to i, and regional heterogeneity analysis based on 10 histone posttranslational modifications by embedding and clustering (iii). **c**, **d** Regional subsets created by clustering as shown in b (iii) were analyzed for 10 different histone posttranslational modifications. Red indicates enrichment, blue indicates depletion. **c** Cluster 1 contains TSS regions of protein-coding genes enriched in marks for actively transcribed chromatin. **d** Cluster 2 contains TSS regions enriched in marks for transcriptionally repressed chromatin. **e**, **f** Chromosome conformation analysis around TSS region subsets as shown in **c**, **d**. **e** HiC average contact maps of cluster 1 from unperturbed wildtype cells (i) and cells depleted of NIPBL (ii). **f** HiC average contact maps as in **e** for cluster 2

To exemplify the power of HiCognition’s region set approach, we analyzed the chromatin fiber organization around all transcriptional start sites (TSS) of protein-coding genes annotated in the human genome [41]. TSS are known to frequently contact upstream and downstream regions; at the same time, TSS insulate against contacts between upstream and downstream genomic regions [42–46]. Using published ChIP-seq data from HeLa cells [8, 47], we first visualized the distribution of two key architectural regulators, cohesin (based on its subunit Structural Maintenance of Chromosomes 3, SMC3) and CCCTC-binding factor (CTCF) using HiCognition’s *1D average widget*. A prominent enrichment of both proteins at TSS (Fig. 2a, panel i) supports a role of cohesin-mediated DNA looping in shaping the conformation around TSS [10, 42, 48, 49].

To assess the 3D organization of protein-coding genes, we next visualized the genome-wide average contact probability around TSS using the *2D average widge*t and published Hi-C data [50] (Fig. 2a, panel ii). Prominent stripes emerging from the TSS towards upstream and downstream regions indicate frequent interactions of TSS with distal genomic regions. Moreover, contacts within regions upstream or downstream the TSS were much more frequent than between upstream and downstream regions (Fig. 2a, visible as red and blue areas, respectively), as previously observed [42–45]. Thus, HiCognition allows simple visualization of genome-wide averages for region-type-specific conformations.

To assess the functional role of cohesin-mediated looping to the conformation at TSS, we next used the *2D average widget* to visualize published Hi-C data obtained from cells depleted of Nipped-B-like protein (NIPBL) [50], a cofactor essential for cohesin-mediated loop extrusion [51, 52] (Fig. 2a, panel iii). The stripes emerging from TSS and the squared regions containing high contact probability that were characteristic for unperturbed controls were almost completely suppressed in the Hi-C maps obtained from NIPBL-depleted cells, indicating a key role of cohesin-mediated looping in establishing these structures, consistent with previous observations [42, 49]. Thus, HiCognition enables fast and interactive side-by-side visualization of genome-wide average profiles across various techniques and experimental conditions.

### Understanding heterogeneity within region sets

Understanding the relationship between chromatin fiber composition, 3D conformation, and physiological function has remained challenging owing to the heterogeneity of regions defined by a common feature under investigation. HiCognition’s region set approach allows fast and simple visualization of regional heterogeneity and supports interactive clustering of these regions based on multiple genomic features.

To demonstrate how HiCognition’s flexible widget architecture can be used for heterogeneity analysis of region sets, we investigated how histone posttranslational modification patterns relate to chromosome conformation around genes. Using the *Stacked lineprofiles widget*, we visualized for the genome-wide set of TSS regions the ChIP-seq read densities of two histone posttranslational modifications, H3K9ac and H3K27me3, which enrich at transcriptionally active or inactive chromatin, respectively [53, 54]. Sorting the line profiles by H3K9ac abundance showed that only about half of the TSS regions were enriched for this mark (Fig. 2b, panel i). Moreover, displaying stacked line profiles of H3K27me3 ChIP-seq read density in a separate widget and sharing the sort order between widgets showed that TSS regions enriched in H3K9ac are depleted of H3K27me3 (Fig. 2b, panel ii). Thus, coupling multiple widgets by sorting allows intuitive visual assessment of correlations between genomic features.

Next, we aimed to identify region subsets with distinct histone modification profiles for the study of the corresponding Hi-C conformations, considering an extended set of ten different histone posttranslational modifications (see the “ Methods” section for details). HiCognition’s *Embedding widget* visualizes regional heterogeneity based on multi-dimensional feature values, which can contain linear profiles such as ChIP-seq data or Hi-C contact matrices (Fig. 2b, panel iii). HiCognition performs dimensionality reduction using Uniform Manifold Approximation and Projection (UMAP, [40]), such that genomic regions that are most similar are displayed in proximity on a two-dimensional map. The genomic regions are then grouped into a user-defined number of clusters based on the similarity of their multi-dimensional feature vectors using the K-means method [55] The *Embedding widget* shows the distribution of all genomic regions and interactively highlights individual clusters of region sets, of which the mean feature values are then displayed either as a bar graph (for one-dimensional epigenetic profiles) or as average maps (for two-dimensional features such as Hi-C contact probabilities). Interactive grouping of multiple clusters allows to the user to freely define new genomic region sub-sets for further analysis. Using the *Embedding widget*, we selected two clusters enriched either in marks for transcriptionally active chromatin or transcriptionally repressed chromatin (Fig. 2b–d) to create two new region subsets for analysis of the corresponding Hi-C conformations.

Using the *2D average widget* and the Hi-C data of HeLa cells, we observed pronounced high-contact stripes and insulation around TSS for the region subset enriched in active chromatin marks, whereas these Hi-C structural features were entirely absent in the region subset enriched in repressive histone marks (Fig. 2e, f, panels i), consistent with previous script-based analyses of mouse stem cell data [42]. To investigate how cohesin-mediated DNA looping contributes to chromosome conformation at TSS residing in transcriptionally active or inactive chromatin, we visualized average Hi-C maps of NIPBL-depleted cells, using published data [50]. For the region subset enriched in transcriptionally active histone marks, we found strong reduction of stripes and insulation around TSS, whereas the region subset with repressive marks was unaffected by NIPBL depletion (Fig. 2e, f, panels ii). Together, these data suggest that cohesin-mediated DNA looping establishes a specific chromosome architecture around transcriptionally active TSS but not at inactive TSS. Thus, HiCognition’s flexible widget architecture enables simple and powerful analysis workflows to explore regional heterogeneity and to detect interactions between different types of genomics data.

### Discovering new associations with HiCognition

Public repositories such as ENCODE [8] or the 4D nucleome [9] contain thousands of different genomics datasets derived from diverse technologies, cell types, and experimental conditions. The difficulty to interpret such complex data has prompted the development of various computational methods to detect associations between specific types of regions and features describing the chromatin fiber, such as GREAT [56], the Encode ChIP-seq significance tool [57], GenometriCorr [58], and Locus Overlap Analysis (LOLA) [39]. HiCognition provides an improved implementation of LOLA, extended by interactive exploration of feature enrichment in distinct genomic sub-bins obtained from a region set. We exemplify association analysis with HiCognition’s *Lola widget* by investigating how cohesin subunit isoforms relate to chromosome conformation.

**Fig. 3:**
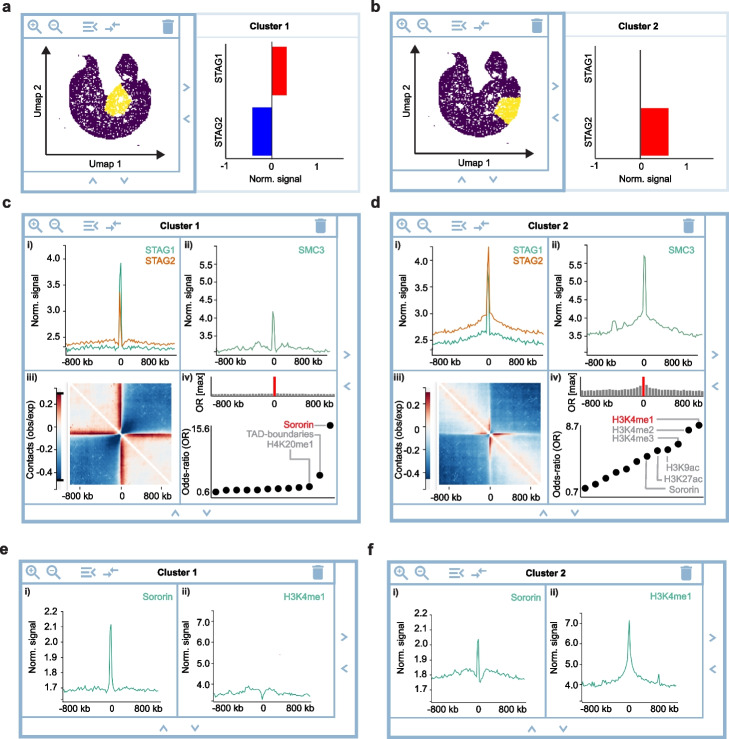
Detecting associations with HiCognition. **a**, **b** Visualization of regional heterogeneity and clustering with the *Embedding widget* for 34,857 SMC3 ChIP-seq peaks based on the ChIP-seq read densities of STAG1 and STAG2. **a** Cluster 1, representing SMC3 ChIP-seq peaks enriched in STAG1. **b** Cluster 2, representing SMC3 ChIP-seq peaks enriched in STAG2 ChIP-seq reads. **c**, **d** Analysis of common patterns and associations for clusters 1 and 2 as in **a**, **b**. **c** Average read density of STAG1 and STAG2 (i) and SMC3 (ii), average Hi-C contact map (iii), and LOLA analysis for associations with 11 region datasets. **d** analysis as in c for Cluster 2. **e**, **f** Validation of Lola analysis by ChIP-seq read density visualization. **e** Average ChIP-seq reads of Sororin and H3K4me1 for the STAG1-enriched cluster 1 as in Fig. **a**, **c**. **f** Average ChIP-seq reads of Sororin and H3K4me1 for the STAG2-enriched cluster 2 as in Fig. **b**, **d**

Cohesin contains three core subunits that form a ring and an associated stromal antigen (STAG) subunit of which vertebrates encode two isoforms, STAG1 and STAG2 [59–62]. Previous script-based analysis of ChIP-seq profiles and Hi-C data showed that STAG2-cohesin predominantly forms loops at active TSS, whereas STAG1-cohesin predominantly contributes to the formation of TADs [60, 63–65]. Here, we aim to recapitulate these findings and search for new associations by the automated machine learning tools and interactive workflows of HiCognition. We created a region set centered on all 34,857 SMC3 ChIP-seq peaks and then clustered SMC3 regions based on the abundance of STAG1 and STAG2, using the *Embedding widget* and published ChIP-seq data [65] (Fig. 3a, b). Comparing ChIP-seq read densities with the *1D average widget* showed that the region subset enriched in STAG1 contained less SMC3 than the region subset enriched in STAG2 (Fig. 3c, d).

To visualize the chromosome conformation around these region subsets, we used the *2D average widget* and published Hi-C data [50]. Strikingly, the STAG1-enriched sites had much more pronounced long-range contacts than the STAG2-enriched sites (Fig. 3c, d, panels iii), despite the lower abundance of the core cohesin subunit SMC3 at STAG1-enriched sites (Fig. 3c, d, panels ii). To determine in which genomic context STAG1- or STAG2-enriched sites predominantly reside, we used the *Lola widget* to analyze 11 region sets including histone posttranslational modifications, TAD boundaries, and the cohesin-associated protein Sororin that is required for cohesion maintenance in G2 [66, 67]. This analysis showed that compared to the genome-wide reference region set, STAG1-enriched sites predominantly reside at TAD boundaries, whereas STAG2-enriched SMC3 peaks predominantly reside in chromatin bearing marks of active transcription (Fig. 3c, d, panels iv), supporting the previously reported distinct localization and function of cohesin bound to STAG1 or STAG2, respectively [60, 63–65]. Moreover, STAG1-enriched cohesin sites also overlapped with Sororin sites detected by ChIP-seq in G2 phase of the cell cycle [47], more prominently than STAG2-enriched cohesin sites, indicating a previously unrecognized association between genomic sites of sister chromatid cohesion and genomic sites where STAG1-enriched cohesin forms long-range loops in G1.

To validate the Lola enrichment analysis, we visualized the most highly scoring features of each cluster, Sororin and H3K4me1, respectively. Average line profiles of ChIP-seq reads show a prominent accumulation of Sororin in the STAG1-enriched cluster, which is less pronounced in the STAG2-enriched cluster (Fig. 3e, f), consistent with the higher odds-ratio calculated by Lola analysis for Sororin in the STAG1-enriched cluster versus the STAG2-enriched cluster (Fig. 3c, d). Conversely, average line profiles of H3K4me1 ChIP-seq reads showed strong accumulation in the STAG2-enriched cluster, but no accumulation in the STAG1-enriched cluster (Fig. 3e, f), again consistent with the odds-ratio values calculated by Lola analysis (Fig. 3c, d).

The region-set-based approach and flexible widget architecture enable detection and validation of such complex associations within a few minutes. HiCognition hence allows biologists untrained in genomic analysis to rapidly perform their own analyses, discover new associations, and generate new hypotheses, greatly reducing the bottleneck between data generation and interpretation.

## Discussion

We present HiCognition as a new visual exploration and machine-learning tool for the detection of patterns and associations between 3D chromosome conformation and collections of 1D genomics profiles. HiCognition’s free public server instance at https://app.hicognition.com/, its rich online documentation, and its containerized distribution supporting desktop as well as server installations provide easy access for both experienced developers as well as beginner analysts. The integrated database and interfaces to widely used file formats allow assessment of a biologist’s own data in the context of the vast amount of public data available from resources like ENCODE or 4D nucleome.

HiCognition’s streamlined workflows and visualization concepts enable users to address a broad range of biological questions, yet the focus on usability limits customizability compared to approaches that simply provide a graphical interface to command-line tools [68] or custom scripts [69]. Via the export of region set coordinates derived from clustering and association analysis, however, HiCognition can be seamlessly integrated with script-based analysis for extended functionality. Hence, HiCognition allows biologists lacking programming skills to rapidly reduce the space of possible hypotheses before applying more time-consuming methods. Furthermore, the software’s modular design and open-source implementation in Python provide an extendable framework towards development of new machine learning algorithms and visualization concepts.

HiCognition will serve as a bridge between the experimentalists who formulate biological hypotheses and specialized computer scientists implementing script-based analyses workflows. This will help biologists understand how the structure and composition of the chromatin fiber contribute to function, particularly when this involves integrated analysis of multiple genomics datasets from various techniques, experimental conditions, and cell states. An integrated analysis of many genomic region sets and feature sets by HiCognition will facilitate the study of diverse processes involving the genome, including transcriptional regulation, DNA repair, and chromosome segregation.

## Conclusion

HiCognition leverages interactive genome exploration to comprehensive views of genome-wide region sets defined by a common property. Its flexible user interface and integrated statistics and machine learning tools support the detection of common patterns, heterogeneity, and associations in complex genomics datasets representing 3D conformation, epigenetic profiles, and functional readouts. A fast and computationally efficient implementation allows real-time browsing through thousands of genomic regions, thereby accelerating hypothesis testing on genomics data of various experimental techniques, experimental conditions, or cell states. While HiCognition’s potential is exemplified here by an analysis of epigenetic marks and topological structures formed by cohesin, the software is applicable to any type of 1D or 2D genomics data. Its ease of use and data integration based on the region set concept will provide new opportunities for discovering relationships between structure and function of the genome.

## Methods

### Software architecture

HiCognition is a containerized application (https://github.com/docker/compose) and designed as a server-client web app to minimize set-up requirements and facilitate easy usage for non-technical users after set-up (Additional file 1: Fig. S1a).

The backend portion of HiCognition is implemented as a Flask webserver (https://github.com/pallets/flask) with NGINX (https://github.com/nginx) as a reverse proxy that operates in conjunction with a MySQL database (https://github.com/mysql) to persist metadata and data preprocessing results. The server utilizes a Redis task queue (https://github.com/rq/rq) to offload time-intensive computation tasks to an adjustable number of worker containers. The communication between these workers and the main server is implemented via network requests (when submitting a task) and the MySQL database (when registering a task as complete). This organization allows the operation of the worker containers on separate machines that could, in principle, be started on demand.

The frontend part of HiCognition is implemented in JavaScript and uses the Vue.js framework (https://github.com/vuejs/vue) to manage components and implement reactivity. The visualizations are custom-designed for each type of data widget (see below for details) and are implemented either using the data-driven visualization library D3.js (https://github.com/d3/d3) or in case of more demanding visualizations using PixiJS (https://github.com/pixijs/pixijs).

For implementation details of the HiCognition architecture, see the GitHub repository (https://github.com/gerlichlab/hicognition) and the accompanying documentation page (https://gerlichlab.github.io/hicognition/docs/).

### Point- and interval-regions

As genomic data frequently span multiple length-scales [16, 17], visualization concepts have to adapt to this challenge. HiCognition solves this problem by precomputing a “resolution-stack” for each genomic region-set (Additional file 1: Fig. S1b). This precomputation is adapted for two types of genomic regions supported by HiCognition:

- *Point-regions* are specified by center coordinates and the region surrounding the center position can be adjusted interactively for analysis and visualization. This enables the user to zoom in and out of genomic regions when viewing data to discover genomic effects at multiple length scales.
- *Interval-regions* are specified by start and end coordinates and each region is then represented as this interval plus 20% neighboring regions on either side. The processing bin size for this region type is automatically adjusted by normalization to the interval size and thus different for differently sized regions. That way, interval regions allow assessment of length-independent patterns, as for example epigenetic profiles around genes of variable length.

### Data management and preprocessing

HiCognition contains a dataset manager that stores available datasets as well as finished pre-computations in a MySQL database. The user interface of HiCognition distinguishes between two principal types of data—genomic regions of interest and genomic features that are available for precomputation (Additional file 1: Fig. S2a). Users can add and view datasets in an interactive table that allows filtering and editing (Additional file 1: Fig. S2b).

HiCognition supports the most common input data formats for genomic regions and features. Specifically, genomic regions can be added as bed-files [15], 1D-features as bigwig files [70] and 2D-features as cooler or hic files [21]. These files can be uploaded one at a time or using a bulk upload feature (see our documentation at https://gerlichlab.github.io/hicognition/docs/data_management/ for details) or directly imported from online repositories, e.g., 4D nucleome [9]) and ENCODE [7, 8], via unique identifier or by providing HiCognition with a weblink.

To analyze a region-set of interest, the user first needs to submit preprocessing tasks using the preprocessing dialogs of the graphical user interface. An overview of running and finished computations is provided via the dataset viewer of the genomic regions (Additional file 1: Fig. S2c). Once pre-computation of a combination of a region-set of interest and a genomic feature has finished, it is available for interactive display using the HiCognition widgets.

Many preprocessing steps involve analysis of genomic feature collections, for example, when calculating enrichment among a set of candidate features or embedding regions based on the values of multiple features (see below for details). In HiCognition, users can create feature collections in a specific dialog window and select them for preprocessing and display.

HiCognition also supports adding and managing multiple genome assemblies to analyze and compare data generated for different genome assemblies and species.

### Data and workflow sharing

HiCognition’s allows storing specific arrangements of widgets, widget collections, and the corresponding data under display as named sessions. This is possible due to an implementation of the HiCognition analysis view as declarative configurations stored in the Vuex frontend storage (https://github.com/vuejs/vuex/). Here, the arrangement, settings, and data sources loaded in a particular widget are stored as JavaScript objects, and HiCognition reacts to changes therein by adjusting the displayed view. This makes it easy to restore saved sessions from configuration objects stored in the database and to share saved sessions with collaborators through a static link.

### Widgets and visualization concepts

HiCognition uses widget-collections as a container to display specific visualizations (Fig. 1b). A widget collection has a single region-set that is shared by all its contained widgets. Each widget in the collection represents a genomic feature or a collection of genomic features and provides a suitable visualization for the respective data (Fig. 1b).

#### 1D-average widget

The 1D-average widget displays the average magnitude of a 1D genomic feature, as for example ChIP-seq reads, for the selected region set in the widget collection as a line plot. The preprocessing algorithm extracts snippets of the relevant genomic feature for each genomic region and calculates the average value over all snippets along the relative genomic offset.

#### 2D-average widget

The 2D-average widget displays the average magnitude of a 2D-genomic feature, for example a Hi-C contact probability map, for the selected region set in the widget collection as a 2D heatmap. The preprocessing algorithm extracts snippets of the 2D-genomic feature for each rectangular genomic region and calculates the average value over all snippets for each pixel.

#### Stacked line profile widget

The stacked line profile widget displays individual examples of 1D-genomic features for the selected region set in the widget collection as a 2D heatmap. Within this heatmap, each row represents a specific genomic region. The preprocessing algorithm extracts the relevant genomic feature snippets for each genomic region (subsampled to contain a maximum of 1000 regions) and “stacks” them vertically to form a matrix for display.

#### 1D-feature embedding widget

The 1D-feature embedding widget displays the distribution of genomic regions based on a collection of 1D genomic features. The results are visualized as a 2D-histogram, where points that are in spatial proximity on the plot represent genomic regions with similar genomic feature profiles.

The preprocessing algorithm extracts the mean signal for all features at every region-set to generate a high-dimensional representation. This representation is a $n x m$ matrix where $n$ is the number of regions in the region-set and $m$ is the number of features used for embedding. To enable visual exploration, the dimensionality reduction algorithm UMAP [40] is used with default parameters to embed the high-dimensional regions into a two-dimensional space suitable for display. Finally, k-means clustering [55] is run to allow the user to easily identify groups of similar regions. The user can select either 10 or 20 regions for the grouping in the widget options. The normalized intensity of the features for each cluster is then calculated and used to interactively display the distribution of features within the selected clusters by mouse hovering. Users can create new region sets from individual clusters, or click on multiple clusters for grouping them into a new customized region set, which can be created and named in the relevant dialog.

#### 2D-feature embedding widget

The 2D-feature embedding widget displays the distribution of genomic regions using a single 2D genomic feature. The results are displayed as a 2D-histogram, where points next to each other represent genomic regions with similar 2D-feature values. The widget provides a hover interaction that shows the 2D average with respect to the selected genomic feature for the selected subset. Users can create new regions from interesting subsets by clicking on a subset and giving it a name in the relevant dialog.

The preprocessing algorithm extracts snippets of the 2D genomic feature for each genomic region in the region set. The goal of this representation is to group together snippets that have high pixel-wise similarity to enable visual exploration of their averages. We utilized a basic feature representation that uses the pixels directly with minimal transformations, as complex image features are not expected to yield interpretable results from snippet averaging. The snippets are first smoothed using a Gaussian filter to remove noise and then down-sampled to a size 10 × 10. The final image size was chosen as a trade-off between preserving information and preventing data scarcity in high-dimensional space for small region-sets. The smoothing kernel size and standard deviation of the Gaussian filter depend on the interpolation factor:

$$I=\left\lfloor\frac{m}{f}\right\rfloor$$

$$K= \left\lfloor\frac{I+1}{2}\right\rfloor$$

$$\sigma =4K+1$$

where $I$ is the interpolation factor, $m$ is the size of the quadratic snippet, $f$ is the target size of the down-sampled matrix (in this case 10), *K* is the size of the smoothing kernel, and $\sigma$ is the standard deviation of the Gaussian filter. The smoothing and down-sampling operations are done using OpenCV (https://github.com/opencv/opencv). Note that since the snippets can be of different sizes (see above for details), the interpolation factor and smoothing function can differ for different extracted snippets. The down-sampled matrix is then flattened and treated as image features for each of the genomic regions, resulting in a matrix where each row corresponds to a genomic region in the region set and each column to one of the pixel features (100 in total). Then, the matrix is embedded into a 2D space using UMAP [40] (https://github.com/lmcinnes/umap), and clustering is performed as for the 1D-feature embedding widget (the user can select either 10 or 20 regions in the widget options). Each cluster is displayed to the user as the 2D average of all contained matrix snippets in the original pixel space.

#### Association widget

The association widget allows users to quantify for a given genomic region set the extent by which other sets of independent genomic regions overlap, based on the LOLA method [39]. As a reference region set for comparison, this analysis always uses all genome-wide bins of matched sizes. This allows to detect associations between different types of genomics data, as for example ChIP-seq peaks and Hi-C structures like boundaries of TADs.

The Association widget provides two visualizations, where the upper bar chart shows for different genomic bins the value of the most highly enriched feature contained in the processed feature set, and the lower chart indicates the enrichment values for all features, in ranked order, for the genomic bin selected by the user.

We reimplementated LOLA [39] (https://github.com/Mittmich/pylola) in Python to improve processing performance, such that the Association widget allows calculating the association not just on the region of interest level but for each individual bin of these regions. Specifically, we use a bin as the target region, the regions in the selected collection as query regions, and all genomic-wide bins of that size as a universe. The reported values correspond to the odds ratio of the underlying contingency table for each combination of target, query, and universe.

### Preparation of datasets for HiCognition

All ChIP-seq data were directly imported into HiCognition based on data from public repositories, except for the SMC3 and Sororin ChIP-seq peaks, which were detected by the following procedure in the published ChIP-seq read profiles from Ladurner et al. [47]:

- Deep (Illumina) sequencing results of ChIP-Seq libraries were downloaded from ENA (ID: SAMEA5988740) and mapped against the human hg19 reference assembly using bowtie resp. bowtie2 (http://bowtie-bio.sourceforge.net/bowtie2/index.shtml) counting only uniquely mappable reads with 0–2 mismatches allowed. Resulting alignments from two replicates each were processed with MACS peak calling algorithm (version 1.4.2) with a *P*-value threshold of 1e − 10 resp. 1e − 5 adding control inputs from the same cell line. Peak overlaps were calculated by using multovl 1.3 (https://github.com/aaszodi/multovl) while treating overlaps as unions and including unique peaks from both replicates. Since occasionally two neighboring peaks from one dataset overlap with a single peak in another dataset, the output of such overlap is displayed as a connected genomic site and merged into one single data entry.
- To derive protein-coding genes split along their direction of transcription, the GENCODE annotations for hg19 (GRCh37) were downloaded and filtered for entries that were of type “gene” and of gene type “protein_coding.” These genes were then split into genes with strand “ + ,” named “forward,” and genes with strand “-,” named “reverse.” The transcriptional start sites for these genes were then defined to be the start or end of these intervals respectively and saved as bed files. The script for this preprocessing step can be found in the HiCognition GitHub repository (https://github.com/gerlichlab/hicognition/blob/master/publication/scripts/convert_genes.ipynb). For the use-case figures, the transcriptional start sites of "forward" oriented genes were used.

### Availability of data and materials

#### Data sources

All datasets used for analysis in the current study have been obtained from public repositories as listed in the following table:

**Table:** 

Name	Repository	ID	Reference
SMC3 Chip-Seq reads	ENA	SAMEA5988740 [71] and SAMEA5988741 [72]	Ladurner et al. [47]
Sororin Chip-Seq reads	ENA	SAMEA3716450 [73] and SAMEA3716449 [74]	Ladurner et al. [47]
CTCF read density	GEO	GSM733785 [75]	Encode Consortium [8]
G2 Hi-C data WT	GEO	GSM4613674 [76]	Mitter et al. [50]
G2 Hi-C data NIPBL depleted	GEO	GSM4613678 [77]	Mitter et al. [50]
Stag1 Chip-Seq read density	GEO	GSM4106803 [78]	Wutz et al. [65]
Stag2 Chip-Seq read density	GEO	GSM4106804 [79]	Wutz et al. [65]
H3K4me1 Chip-Seq peaks	GEO	GSM798322 [80]	Encode Consortium [8]
H3K4me2 Chip-Seq peaks	GEO	GSM733734 [81]	Encode Consortium [8]
H3K4me3 Chip-Seq peaks	GEO	GSM733682 [82]	Encode Consortium [8]
H3K9me3 Chip-Seq peaks	GEO	GSM1003480 [83]	Encode Consortium [8]
H3K9ac Chip-Seq peaks	GEO	GSM733756 [84]	Encode Consortium [8]
H3K79me2 Chip-Seq peaks	GEO	GSM733669 [85]	Encode Consortium [8]
H3K27ac Chip-Seq peaks	GEO	GSM733684 [86]	Encode Consortium [8]
H4K20me1 Chip-Seq peaks	GEO	GSM733689 [87]	Encode Consortium [8]
H3K36me3 Chip-Seq peaks	GEO	GSM733711 [88]	Encode Consortium [8]
H3K27me3 Chip-Seq peaks	GEO	GSM733696 [89]	Encode Consortium [8]
H3K27me3 Chip-Seq read density	GEO	GSM733696 [89]	Encode Consortium [8]
H3K9me3 Chip-Seq read density	GEO	GSM1003480 [83]	Encode Consortium [8]
H3K4me3 Chip-Seq read density	GEO	GSM733682 [82]	Encode Consortium [8]
H3K9ac Chip-Seq read density	GEO	GSM733756 [84]	Encode Consortium [8]
H3K36me3 Chip-Seq read density	GEO	GSM733711 [88]	Encode Consortium [8]
H3K79me2 Chip-Seq read density	GEO	GSM733669 [85]	Encode Consortium [8]
H3K27ac Chip-Seq read density	GEO	GSM733684 [86]	Encode Consortium [8]
H4K20me1 Chip-Seq read density	GEO	GSM733689 [87]	Encode Consortium [8]
H3K4me2 Chip-Seq read density	GEO	GSM733734 [81]	Encode Consortium [8]
H3K4me1 Chip-Seq read density	GEO	GSM798322 [80]	Encode Consortium [8]
gencode.v38lift37.basic.annotation.gtf	GENCODE	Release38 GRCh37 [90]	Gencode Project [41]
G2 Hi-C data WT TADs	GitHub	TADs_final.bedpe [91]	Mitter et al. [50]

#### Codebase

HiCognition is an open-source MIT licensed project and as such, we welcome all contributions to our codebase. To facilitate external contributions to the HiCognition project, the source code is well documented, and we provide extensive unit and integration tests for all components. The code is maintained at GitHub (https://github.com/gerlichlab/hicognition) [92] and will be continuously expanded and updated. Users are welcome to request features and improvements directly via GitHub issues. The version presented in this article is 0.7 and available as a Zenodo archive (https://zenodo.org/record/7972857) [93].

#### Installation

HiCognition is a containerized application that can be installed on local machines in three steps: First, clone the repository from GitHub, then configure the environment variables if necessary to local needs, and start the server with a single command “docker-compose up –d.” After that, HiCognition is available on port 80 on your local machine, and any web browser that has access to this port can utilize it (Chrome browser is recommended). For a more in-depth description of the installation procedure, visit: https://hicognition.com/docs/installation/.

#### Public server

To provide readers a fast hands-on experience of HiCognition, we provide a public server (https://app.hicognition.com/). This server has all functionality enabled and users can sign up for a free account using a valid e-mail address. We uploaded and preprocessed all the datasets in this paper so the reader can explore them independently. In addition, readers can upload their own data and preprocess them for detailed exploration.

## Supplementary Information

**Additional file 1: Fig. S1**. Diagrams depicting the implementation of HiCognition. **Fig. S2.** Explanation of the user interface for dataset management.

**Additional file 2. **Review history.
