# Supplementary material for: HiCognition: a visual exploration and hypothesis testing tool for 3D genomics
Source: Genome Biol. 2023 Jul 5;24:158. doi: 10.1186/s13059-023-02996-9 (PMC10320903; doi:10.1186/s13059-023-02996-9)
Supplement: Supplementary file 1 — Additional file 1: Fig. S1. Diagrams depicting the implementation of HiCognition. Fig. S2. Explanation of the user interface for dataset management. [file 13059_2023_2996_MOESM1_ESM.pdf]

Fig. S1

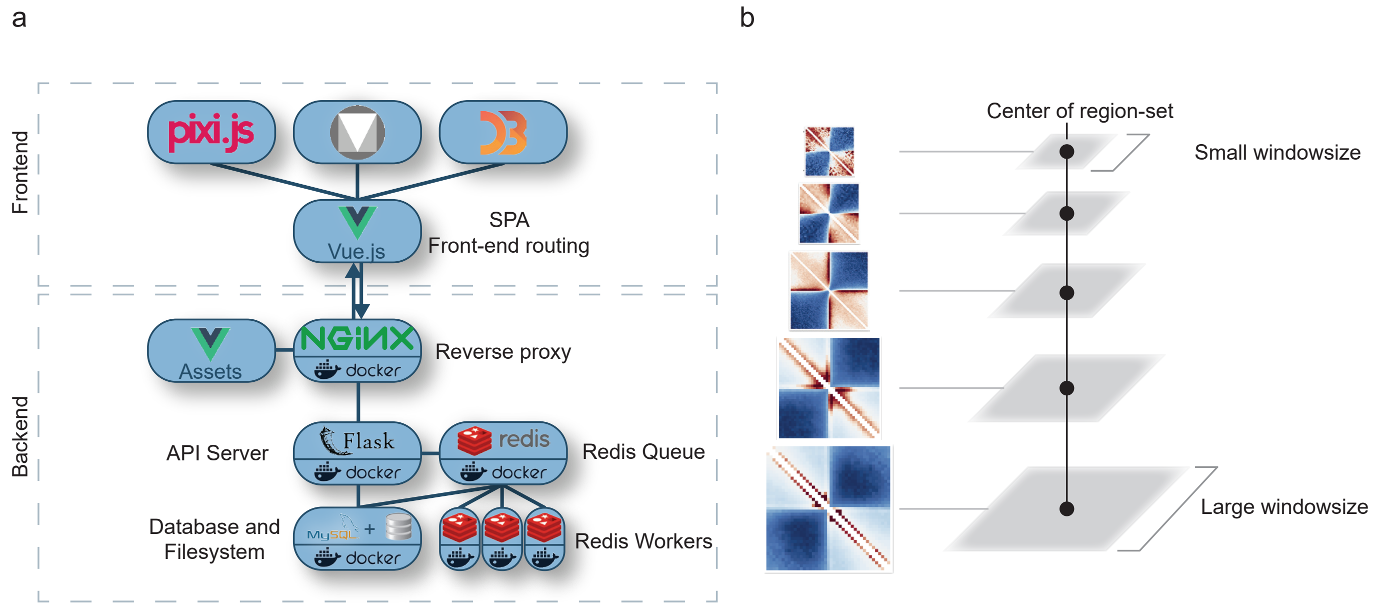

Supplementary Figure 1. Implementation of HiCognition. a, HiCognition is a single-page application connected to an API backend server. The backend components are orchestrated and containerized via docker and consist of a NGINX web server, the Flask API server that dispatches high-performance precomputation tasks into a Redis queue, and a MySQL database for persistence. The frontend is based on the Vue.js JavaScript framework. The user interface is built with “Vue Material” components. The custom visualizations are built with the d3.js visualization library and the pixi.js rendering library. **b,** HiCognition precomputes a resolution stack with different window sizes and resolutions around a genomic region set to allow real-time exploration of its multi-scale neighborhood.

Fig. S2

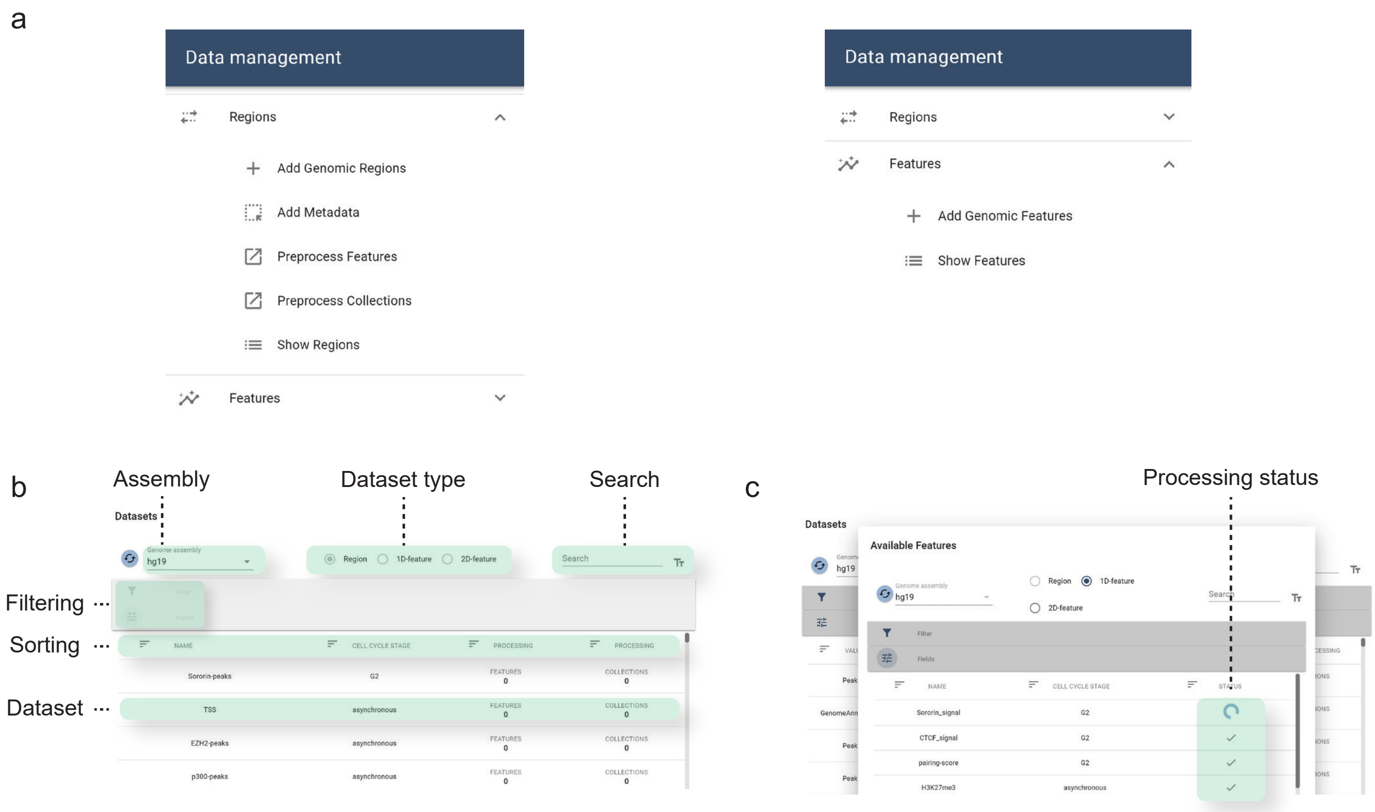

Supplementary Figure 2. User interface for data set management. a, HiCognition provides conceptual separation between genomic regions of interest and genomic features. This is captured in the data management functionality by separating the options to upload, preprocess and edit genomic regions and features. **b,** HiCognition provides an interactive dataset table for managing genomic datasets. This includes selecting genome assemblies, filtering on metadata, searching for datasets, and modifying and deleting datasets. **c,** Within the dataset table, the processing state of genomic features for a specific genomic region set can be viewed within a processing dialogue.
