# Supplementary material for: HiCognition: a visual exploration and hypothesis testing tool for 3D genomics
Source: Genome Biol. 2023 Jul 5;24:158. doi: 10.1186/s13059-023-02996-9 (PMC10320903; doi:10.1186/s13059-023-02996-9)
Supplement: Supplementary file 2 — Additional file 2. Review history. [file 13059_2023_2996_MOESM2_ESM.docx]

Review History

**First round of review**

**Reviewer 1**

**Were you able to assess all statistics in the manuscript, including the appropriateness of statistical tests used?**

There are no statistics in the manuscript.

**Were you able to directly test the software?**

No.

**Comments to author:**

In the manuscript GBIO-D-22-00972, Langer et al presented Hicognition, a visual navigation tool for 3D genome. The visual exploration in Hicognition is centered on a region-set of interest defined by the users. For any given region set, Hicognition can carry out three types of data analysis and visualize them simultaneously: 1) the average distributions of the selected genomic signals; 2) the heterogeneity of the selected genomic signals in the specific region set; and 3) associated features that are enriched or depleted in the specific region set. If fully developed, Hicognition can be very useful to the field. However, it seems that Hicognition is still at its very early stage of development.

Major concerns:

1. The region-set focus approach is the core concept of Hicognition that distinguishes it from other existing genome browsers. However, at the current setting, it requires users to define the region set of interest by providing a file containing genome coordinates as input data, which is practically demanding for the ordinary biologists without programming skills. --- One way to overcome this limitation might be that Hicognition can pre-build an index library that contains the various popular region sets of interest and allow the users to choose from them.

2. The defining of sub-regions can be more flexible. According to figures 2 and 3, sub-regions are defined by unsupervised clustering. It will be very helpful if Hicognition can allow users to select any kind of subregions freely.

3. It will be very helpful if Hicognition can allow simultaneously visualize and explore a more diversity of multimodal data types, as the Nucleome Browser does.

4. Hicognition currently allows desktop installation, however, the installation process for a regular user is not as simple as the authors thought. It will be more helpful if a web-based platform can be provided.

**Reviewer 2**

**Were you able to assess all statistics in the manuscript, including the appropriateness of statistical tests used?**

There are no statistics in the manuscript.

**Were you able to directly test the software?**

Yes.

**Comments to author:**

This paper introduces a software tool for visualizing and aggregating genomic information over disparate regions. The software is nicely documented. It is implemented in Docker, which makes it easy to install on any system. The design is attractive and the widget aspect allows for flexibility. The concept of a UMAP between 1D and 2D tracks seems interesting (with some caveats below). I did access the demo and play around a little bit; though I found the demo overall unintuitive, I appreciate the effort to create a useful demonstration. The authors also showed a relevant biological discovery enabled by their software, though this was a bit hidden.

I do have several questions and concerns about the utility of the software. One must run their own server and transfer files in order to be preprocessed. This takes a lot more work than your standard genome browsers, which operate entirely on the web on streaming data. To this point, the software does not seem to support the streamable ".hic" format for 2D analysis. ENCODE has released over 100 Hi-C experiments in this file format; one would have to reprocess something into a different format to be accessible in this genome browser, whereas a genome browser like WashU can directly load these files from ENCODE. Also, at the end of the paper the authors say this can be integrated with script-based analysis for extended functionality, but how to do this was not obvious.

The analysis portion seems weak. The authors name three tasks: finding the average distribution over a genomic region, finding differences, and enrichment analysis. The first seems useful if trivial. The second introduces the idea of interactive cluster analysis across regions. This idea is intriguing but lacking in a mathematical foundation / explanation. As it is not a standard analysis tool, it needs to be explained and justified; moreover, this portion is less useful if the community as a whole does not normally perform this kind of analysis. Finally, enrichment analysis is useful and widely accepted, but it was not clear what the enrichment is relative to (the genome-wide average? within the region set?). False positives are an ever-present problem with 2D enrichment tools. Does choosing the regions in this way skew the enrichment analysis tool? Seeing some visual examples showing that the enrichment is real would be helpful; proving it is via statistics (e.g. FDR) would be even better. Overall, the claims of "integrated statistics and machine learning tools" seem overblown.

**Authors Response**

**Point-by-point responses to the reviewers’ comments:**

*Response to the reviewers*

*We thank both reviewers for their thoughtful comments and constructive suggestions how to improve the manuscript and the HiCognition software. We addressed all their comments as explained point-by-point below. The most important revisions are:*

*• Implementation of a free public server instance of HiCognition, so that the software can be used without the need for local installation*

*• Improved usability of HiCognition by supporting direct import of genomic region set and feature data from public repositories, including ENCODE and 4D Nucleome*

*• More versatile chromosome conformation analysis by supporting “.hic”, “.cooler”, and “.bedpe” file formats*

*• Free selection of genomic sub-regions by interactive grouping of smaller clusters*

*• More detailed explanation of the dimensionality reduction and clustering methods*

*• More detailed explanation and validation of the Lola enrichment analysis*

Reviewer #1

In the manuscript GBIO-D-22-00972, Langer et al presented Hicognition, a visual navigation tool for 3D genome. The visual exploration in Hicognition is centered on a region-set of interest defined by the users. For any given region set, Hicognition can carry out three types of data analysis and visualize them simultaneously: 1) the average distributions of the selected genomic signals; 2) the heterogeneity of the selected genomic signals in the specific region set; and 3) associated features that are enriched or depleted in the specific region set. If fully developed, Hicognition can be very useful to the field. However, it seems that Hicognition is still at its very early stage of development.

Major concerns:

1. The region-set focus approach is the core concept of Hicognition that distinguishes it from other existing genome browsers. However, at the current setting, it requires users to define the region set of interest by providing a file containing genome coordinates as input data, which is practically demanding for the ordinary biologists without programming skills. --- One way to overcome this limitation might be that Hicognition can pre-build an index library that contains the various popular region sets of interest and allow the users to choose from them.

*Reply: We appreciate the suggestion to further improve the usability of HiCognition by facilitating access to region set data. As most region sets are cell-type- and context-specific, we do not think that a pre-built index library would be a suitable solution. However, to address this concern, we extended HiCognition so that via its graphical user interface genomic region datasets can be directly imported from public repositories, including 4D Nucleome and ENCODE. This facilitated import functionality will allow users to easily find, access, and add various well-curated and annotated genomic region sets of interest. Detailed instructions how to import genomic region data sets from public repositories are provided in the online software documentation, see https://hicognition.com/docs/data_management/.*

2. The defining of sub-regions can be more flexible. According to figures 2 and 3, sub-regions are defined by unsupervised clustering. It will be very helpful if Hicognition can allow users to select any kind of subregions freely.

*Reply: We agree that a more flexible selection of sub-regions would make the analysis more versatile. We therefore extended HiCognition so that the user can now calculate a larger number of clusters and then freely group these smaller clusters into larger sub-regions by interactive selection.*

3. It will be very helpful if Hicognition can allow simultaneously visualize and explore a more diversity of multimodal data types, as the Nucleome Browser does.

*Reply: We would like to clarify that HiCognition can simultaneously visualize many different data types, including chromosome conformation contact maps (e.g., from Hi-C or SPRITE, protein binding profiles (e.g., ChIP-seq or Cut&Run read densities), chromatin accessibility measurements (e.g., ATAC-seq or MNase-seq), transcriptional activity (e.g., GRO-seq), or replication timing measurements (e.g., Repli-seq). We now also extended HiCognition by the support of 2D regions via .bedpe files. Thus, most of the data types that can be displayed in the Nucleome Browser can now also be displayed in HiCognition. The diverse data types that can be visualized with HiCognition are listed in the main text.*

4. Hicognition currently allows desktop installation, however, the installation process for a regular user is not as simple as the authors thought. It will be more helpful if a web-based platform can be provided.

*Reply: We agree that a web-based platform will facilitate usage of HiCognition. We therefore implemented a fully web-based platform that is freely accessible at https://app.hicognition.com. Any interested user will be able to register with their email address upon publication of the manuscript to benefit from the full functionality of HiCognition without any setup. We also streamlined the installation process and provide more detailed instructions online for those users who would like to install a local instance for improved upload speeds via local networks.*

*To enable anonymous testing of the HiCognition server instance (https://app.hicognition.com), we generated the following login credentials for temporary usage during the peer-review process:*

*Username: reviewer1*

*Password: t*03K8no*

Reviewer #2

This paper introduces a software tool for visualizing and aggregating genomic information over disparate regions. The software is nicely documented. It is implemented in Docker, which makes it easy to install on any system. The design is attractive and the widget aspect allows for flexibility. The concept of a UMAP between 1D and 2D tracks seems interesting (with some caveats below). I did access the demo and play around a little bit; though I found the demo overall unintuitive, I appreciate the effort to create a useful demonstration. The authors also showed a relevant biological discovery enabled by their software, though this was a bit hidden.

I do have several questions and concerns about the utility of the software. One must run their own server and transfer files in order to be preprocessed. This takes a lot more work than your standard genome browsers, which operate entirely on the web on streaming data.

*Reply: We agree that a free public server instance of HiCognition and direct import of data from public repositories would improve usability. We therefore implemented and launched a public webserver at https://app.hicogntion.com. Upon publication of this manuscript, will be able to register with their email address upon publication of the manuscript to benefit from the full functionality of HiCognition without any setup. We also extended HiCognition so that via its graphical user interface genomic region datasets can now be directly imported from public repositories, including 4D Nucleome and ENCODE. These improvements will allow users to easily find, access, and add various well-curated and annotated genomic region sets of interest, without the need to install a local version of the software.*

*To enable anonymous testing of the HiCognition server instance (https://app.hicognition.com), we generated the following login credentials for temporary usage during the peer-review process:*

*Username: reviewer2*

*Password: at22^Z5#*

To this point, the software does not seem to support the streamable ".hic" format for 2D analysis. ENCODE has released over 100 Hi-C experiments in this file format; one would have to reprocess something into a different format to be accessible in this genome browser, whereas a genome browser like WashU can directly load these files from ENCODE. Also, at the end of the paper the authors say this can be integrated with script-based analysis for extended functionality, but how to do this was not obvious.

*Reply: We thank the reviewer for the suggestion to enhance the versatility of the software by supporting additional file formats. HiCognition now supports “.hic” and also “.cooler” formats for Hi-C experiments. Moreover, HiCognition can now also directly import data from ENCODE and 4D Nucleome via its graphical user interface. The detailed instructions for data import are provided in the online documentation, see https://hicognition.com/docs/data_management/*

The analysis portion seems weak. The authors name three tasks: finding the average distribution over a genomic region, finding differences, and enrichment analysis. The first seems useful if trivial.

*Reply: The strength of HiCognition is to provide an integrated solution for the interactive analysis of large genomic region sets and multi-dimensional feature sets. While visualizing the average distribution of features is mathematically straightforward, it is an important element in more complex and workflows using more advanced functionalities of HiCognition, such as visualization of regional heterogeneity by dimensionality reduction, clustering, and locus overlap analysis. We now showcase how the visualization of average distributions can be used to validate Lola enrichment analysis results (see below and new Fig. 3e, f).*

The second introduces the idea of interactive cluster analysis across regions. This idea is intriguing but lacking in a mathematical foundation / explanation. As it is not a standard analysis tool, it needs to be explained and justified; moreover, this portion is less useful if the community as a whole does not normally perform this kind of analysis.

*Reply: We thank the reviewer for pointing out the need to better explain the cluster analysis across regions and extended the main text and methods sections accordingly. We visualize the data by first transforming multi-dimensional feature vectors associated with each genomic region (e.g., ChIP-seq, ATAC-seq, Hi-C, etc.) into an embedded two-dimensional representation using Uniform Manifold Approximation and Projection (UMAP). We cluster the data points using the K-means method and plot them in the widget. For each of the clusters, HiCognition interactively displays the mean of individual feature values as a bar graph (for one-dimensional features) or an average map (for two-dimensional features), so that the user can explore the specific properties of region sub-sets contained in each cluster. The mathematical foundation of K-means clustering and data visualization of multi-dimensional data by Uniform Manifold Approximation and Projection (UMAP) is well established and appropriate references for these methods are now cited in the methods section.*

*We respectfully disagree that providing a technique that is not yet widely used in the community as part of an interactive data exploration tool represents limited relevance. In contrast, we think that easy access to such new techniques via a graphical user interphase has particularly high potential to innovate data analysis workflows on complex multi-dimensional genomics data.*

Finally, enrichment analysis is useful and widely accepted, but it was not clear what the enrichment is relative to (the genome-wide average? within the region set?). False positives are an ever-present problem with 2D enrichment tools. Does choosing the regions in this way skew the enrichment analysis tool? Seeing some visual examples showing that the enrichment is real would be helpful; proving it is via statistics (e.g. FDR) would be even better. Overall, the claims of "integrated statistics and machine learning tools" seem overblown.

*Reply: We thank the reviewer for pointing out that the enrichment analysis should be better explained and validated. In HiCognition, the enrichment is calculated relative to the genome-wide average, which we now state in the main text and methods section. We note that the enrichment analyses is performed not only at the central bin of the genomic region set, but also at neighboring genomic regions, as displayed by a bar plot in the Lola widget. Hence, the user can visually assess the degree of enrichment in the central bin relative to the neighboring genomic regions. These methods details are now explained in the Methods section.*

*To formally address the concern raised by the reviewer, we extended Figure 3 by showing the ChIP-seq profiles of the two features predicted by the Lola widget to have highest overlap with each of the target regions contained in the two clusters (Sororin for the STAG1-enriched cluster, and H3K4me1 for the STAG2-enriched cluster). Average line profiles of ChIP-seq reads show a prominent accumulation of Sororin in the STAG1-enriched cluster, which is less pronounced in the STAG2-enriched cluster (Fig. 3e, f), consistent with the higher odds-ratio calculated by Lola analysis for Sororin in the STAG1-enriched cluster versus the STAG2-enriched cluster (Fig. 3c, d). Conversely, average line profiles of H3K4me1 ChIP-seq reads showed strong accumulation in the STAG2-enriched cluster, but no accumulation in the STAG1-enriched cluster (Fig. 3e, f), again consistent with the odds-ratio values calculated by Lola analysis (Fig. 3c, d).*

**Second round of review**

**Reviewer 1**

The authors have fully addressed my concerns raised in the previous round.

**Reviewer 2**

The authors have addressed many of my concerns. I have tried to use their demonstration and still found it unintuitive; some things that would help:

- Documentation that shows how to load from external databases (ENCODE, 4DN); I looked at the documentation but it was not obvious. In UCSC / IGV / Epigenome, it's very easy to load directly from ENCODE; it seemed like I needed to first search the URL in order to do so in this tool, which is not straightforward. In an ideal world, there would be an API call and you could simply select from ENCODE datasets that match your search terms

- Similarly, when I clicked on datasets, it was often unclear where the datasets came from (Description says "No description provided"). Sometimes there is a notation; all of these should be annotated, particularly since the examples are coming from this paper and thus you have the provenance.

- Some common assemblies should be standard and not need upload - e.g., hg38, mm10. Again this step is already done for you in other genome browser tools (for common genomes). It still seems like lots of things need to be uploaded and this is going to be hard for someone not versed in bioinformatics (the intended audience).

**Authors Response**

**Point-by-point responses to the reviewers’ comments:**

Reviewer #1:

The authors have fully addressed my concerns raised in the previous round.

Reply: We thank the reviewer for the positive assessment of our revised manuscript.

Reviewer #2:

The authors have addressed many of my concerns. I have tried to use their demonstration and still found it unintuitive; some things that would help:

- Documentation that shows how to load from external databases (ENCODE, 4DN); I looked at the documentation but it was not obvious. In UCSC / IGV / Epigenome, it's very easy to load directly from ENCODE; it seemed like I needed to first search the URL in order to do so in this tool, which is not straightforward. In an ideal world, there would be an API call and you could simply select from ENCODE datasets that match your search terms.

*Reply: We extended the documentation and now provide step-by-step instructions, including detailed example pictures. We now also include an icon next to the input field that will open a second window to the search site of the corresponding repository. Here, the user can freely use all the available search filters and detailed inspection tools specifically designed for the different repositories or follow associated external links to find the correct match for the user's need. This software update makes the workflow more streamlined and less dependent on unique constraints of older or up-and-coming repositories APIs and lets us stay as general as possible.*

- Similarly, when I clicked on datasets, it was often unclear where the datasets came from (Description says "No description provided"). Sometimes there is a notation; all of these should be annotated, particularly since the examples are coming from this paper and thus you have the provenance.

*Reply: We thank the reviewer for pointing out the missing metadata for our example datasets. This information was by mistake omitted during our paper revision, when we migrated data to the public* *server. We now added the missing annotation to the datasets provided by us on the public server for HiCognition.*

- Some common assemblies should be standard and not need upload - e.g., hg38, mm10 Again this step is already done for you in other genome browser tools (for common genomes). It still seems like lots of things need to be uploaded and this is going to be hard for someone not versed in bioinformatics (the intended audience).

*Reply: We appreciate the suggestion to upload more standard genome assemblies. We added hg38, mm9, mm10 and sacCer3 to hg19. We further updated the documentation to include examples of finding or generating the necessary files if a user needs to upload other genome assemblies.*
